# The effect of geography and climatic factors on the hospitality burden due to kidney stones in Spain 1997-2022.

**DOI:** 10.12688/openreseurope.23359.2

**Published:** 2026-07-15

**Authors:** Javier Saenz-Medina, Sara Garach-Fernández, Ana Tagalos-Muñoz, Victoria Gómez Dos Santos, Manuel Hevia-Palacios, Ana Dominguez-Gutierrez, Laura Prieto-Matienzo, Federico Soria Gálvez, Francisco Javier Burgos-Revilla

**Affiliations:** 1 Hospital Universitario Ramon y Cajal. Servicio de Urología, Madrid, Community of Madrid, Spain; 2 Universidad Rey Juan Carlos Especialidades Medicas y Salud Publica, Madrid, Community of Madrid, Spain; 3 Hospital Universitario Ramon y Cajal Servicio de Cirugía experimental, Madrid, Community of Madrid, Spain

**Keywords:** kidney stones; climatic factors; geographical factors, epidemiology.

## Abstract

The incidence of kidney stone disease (KSD) has been rising in the last decades, becoming a worldwide problem. Besides dietary factors, climatic conditions have also been identified as possible reasons for this.

Our objective was to describe the hospitality burden due to KSD across different regions of Spain and relate it to different climatic conditions.

**Methods:**

An observational retrospective study was conducted during the period 1997–2022 by analyzing hospitalization records from the minimum basic dataset. The data were related to climatic conditions (temperature, humidity, evaporation, and solar radiation) and were collected from the Spanish Meteorological Agency.

**Results:**

A total of 980701 hospitalizations were collected, estimating a burden of 8.4 patients per 10000 inhabitants (CI95%: 8.4–8.4) in this period. A significant increasing trend with an annual percentage change of 2.14% (CI95%: 0.9–3.4), was observed in this period. Hospitalizations were higher in men 9.6 patients per 10000 inhabitants (CI95%: 9.6–9.7) than in women (8.2, CI95%: 8.2–8.3).

Humidity was the only factor that reached a significant inverse correlation (r = −0.26(CI95%: −0.26, −0.26), keeping itself as an independent factor when linear regression was applied. The temperature, radiation, and evaporation were not statistically significant. A higher risk of hospitalization for KSD in spring (OR: 1.1; CI95%: 1.1–1.1) was also observed.

**Conclusions:**

Our study confirms a significant rising trend of KSD hospitalizations with significant differences across different regions, which could be partially explained by changes in humidity values. Temperature, solar radiation, and evaporation did not seem to have any influence on KSD hospitalizations.

## Introduction

An increasing incidence of kidney stones has been reported in developed countries in the last decades of the past century. Although dietary factors have been identified as one of the most important reasons, other factors, such as climatic conditions or socioeconomic status, have also been related to kidney stone formation. ^1^

Several environmental factors are associated with kidney stone formation. Regarding climatic factors, it has been well documented that the increase in global temperatures observed since 1850 can contribute to an increase in kidney stone disease (KSD) that has occurred in parallel. Additionally, KSD has been documented to be more prevalent in lower latitude locations, such as southern America and southeast Asia. ^2, 3^ Occupational groups exposed to higher temperatures have also shown a higher KSD risk, although no systematic review has been performed regarding this issue. ^3^

From a pathophysiological point of view, the mechanisms behind this relationship are not clear enough, nor are the roles of particular climatic elements, such as temperature, humidity, evaporation, or sunlight. Higher transdermal insensible losses through breathing or sweating can result in the supersaturation of urinary solutes and promotion of urinary crystallization. ^4^ Although the role of humidity in promoting stone disease is inconclusive, it has been suggested that temperature combined with humidity, via the wet-bulb temperature, can be associated with KSD. ^5^ Regarding the relationship to climatic conditions, a higher KSD risk for men than for women has been reported for the same temperatures, suggesting sexually different physiological responses to exposure to ambient temperatures. ^6^

Additionally, heat stress has also been physiologically associated with acute kidney injury (AKI) since heat leads to increases in vasopressin secretion and activation of the renin-angiotensin-aldosterone axis due to extracellular fluid volume loss. Renal artery vasoconstriction and decreased cortical blood flow trigger a reduction in O _2_ supply and ATP. Hyperosmolarity also leads to an increase in the polyol-fructokinase pathway, resulting in an increased amount of fructose, causing oxidative stress and inflammatory responses, which are extensively associated with urinary stone disease (USD). ^7^

Spain’s diverse climate can be broadly described as having a Mediterranean influence, with hot, dry summers and mild, wet winters, particularly in coastal areas. However, the country shows significant regional variations. Spain offers a wide range of weather conditions, depending on the specific location and time of the year. In Spain, a survey-based study conducted on over 5000 people observed a significant correlation between the prevalence of KSD and the annual average temperature in different regions. ^8^

In this study, we aimed to investigate the hospitality burden due to kidney stone disease over different regions in Spain and to relate it to different climatic conditions in order to establish how temperature, humidity, evaporation, or solar radiation can influence KSD prevalence.

## Methods

### Study design

A retrospective observational study of Spanish hospitalizations based on the Minimum Basic Data System (MBDS) was performed. MDBS is a public registry of the Ministry of Health and Social Services for both public and private hospitals and covers 98% of the total hospitalizations. It provides data on hospital morbidity classified by region along with other data such as procedures performed, length of hospitalization, and demographic data. The ICD-9 MC (1997–2015) and ICD 10-MC (2016-nowadays) classifications are used for diagnosis codes. All the information used by the researchers was coded and respected anonymously.

All hospitalizations from 1997 to 2022 that include in diagnostic code “ureteral, or kidney stone” have been collected. The ICD-9MC and ICD-10 MC codes are presented in Table 1.

**Table 1.** ICD9-MC and ICD10-MC diagnostic codes used for the study.

	CIE9 MC	CIE10 MC
Description	1997–2015	2016–2021
**Diagnostic codes**		
Ureteral stone	592.1	N20.1
Kidney stone	592.0	N20.0
Kidney and ureteral stone		N20.2
Hydronephrosis with renal and ureteral stone obstruction	591	N13.2
Non specified urinary stone	592.9	N20.9

Hospitalization rates were calculated per 10000 inhabitants, according to the information of the general population per region of the National Institute of Statistics. ^9^

Spain comprises 17 autonomous regions and two autonomous cities (Ceuta and Melilla). Autonomous regions are territorial entities with political legislative and administrative autonomy, although they are also subject to the Spanish Constitution. Spain’s administrative division into administrative regions is based on a country’s historical and cultural regional organization. Meteorogical variables per region from 2016 to 2022 are available on the web page of the Spanish Meteorological Agency. ^10^ Average temperature, humidity, radiation, and evaporation have been calculated per year and by region, and have been analyzed in relation to regional hospitalization rates of urinary Stone disease by year.

### Statistical analysis

A descriptive analysis of the kidney stone hospitalization rate from 1997 to 2022 was performed for each region. Data are presented per 10000 population with a 95% confidence interval. For trend estimation, a join-point regression model was used to calculate the annual percentage change. using the log transformation of age-standarized rates with its correspondant 95% CI. In our model, minimum number of joinpoints are zero, and maximum number number of joinpoints are four. The method used to select the final model was the weighted Bayesian information criterion. Finally, the model recommended was with zero joinpoints.

To establish the influence of temperature, humidity, radiation, and evaporation on hospitalization burden for kidney stone disease, a Pearson correlation test was performed between the average measures and hospitalization burden from 2016 to 2022 per year and region. No meteorological data were available from 1997 to 2015 from the Spanish meteorogical agency. In addition, a correlation between the average climatic factors (temperature, humidity, evaporation, and radiation) of each region and kidney stone disease burden hospitalization was performed to determine if the differences between regions were explained by climatic factors. In addition, average climatic conditions were related to annual burden to explain if the changes over the years could have repercussions in the hospitalization of patients with KSD. Choropleth maps have been performed to visualize the distribution of the meteorological variables across the country (Suppl file 1).

Multivariate analysis through linear regression was performed to establish the influence of humidity and temperature on hospitalization burden due to kidney stones.

In addition, a comparative analysis was performed using an ANOVA test to establish the influence of season on hospitalization burden for each region. All tests were performed using SPSS V.24 statistical software package.

## Results

### Demographic and basal characteristics

A total of 980701 hospitalizations with a diagnosis of kidney or ureteral stones from 1997 to 2022 were recorded in Spain, according to MDBS data. The mean age of the patients was 58.9 years (CI 95%: 58.9–58.9); 552432 patients (56.3%; CI95%; 56.2–56.4) were male, and 428194 patients (43.7%, CI95%: 43.6–43.8) were female; in 75 patients, the sex was not recorded. The mortality rate of Spanish hospitalizations for kidney stones was 2.9% (CI95%: 2.9–3) and the length of stay was 7.2 days (CI95%: 7.2–7.3 days).

The average temperature in Spain from 2016 to 2022 was 16.02 °C (CI95%: 15–17.04). The minimum average temperature was registered in Castilla y León (13 °C) and the highest in Murcia (19.2 °C). The average humidity was 64.4% (CI95%: 61.4–67.4), registering the lowest average in Murcia (57.3%) and the highest in Asturias (74.6%). Average radiation was 49439 wh/m ^2^/day (49364.8–49632.6), registering the lowest average radiation in Navarra (37376.5 wh/m ^2^/day), and the highest in Baleares (63711.9 wh/m ^2^/day). Average evaporation was 1487.4 mm (95%CI: 1276.6–1698.1), lowest average evaporation was registered in Asturias 831.4 mm and the highest in Madrid 2112.3.

### Trends of hospitalizations of urinary stone disease per region

It has been estimated the overall hospitalization burden of 8.4 patients per 10000 inhabitants (CI95%: 8.4–8.4) in Spain from 1997 to 2022. Castilla y León, a continental region located in the northern part of the Spanish central plateau, presented the highest incidence of hospitalization for urinary stone disease reaching 11.3 patients per 10000 inhabitants followed by Murcia, Cataluña, Extremadura, and Aragón. Conversely, the Canary Islands had the lowest number of patients (CI95%: 4.1–4.4) followed by Melilla, Andalucía, and Cantabria ( Table 2 and Figure 1).

**Table 2.** Five-year rates of hospitalization burden of patients with kidney or ureteral stones between 1997 and 2022 stratified by region. Data are given as a rate (95% ci) per 10000 inhabitants.

Region	1997–2001	2002–2006	2007–2011	2012–2016	2017–2022	Overall 1997–2022
1.- Andalucía	5.7 (5.6–5.8)	6.6 (6.6–6.7)	5.8 (5.7–5.8)	7.3 (7.3–7.4)	8.6 (8.6–8.7)	6.9 (6.9–7)
2.- Aragón	10.2 (10–10.5)	12.5 (12.3–12.8)	9 (8.8–9.2)	10.8 (10.6–11.1)	10.9 (10.7–11.2)	10.7 (10.6–10.8)
3.- Asturias	7.9 (7.7–8.1)	9.1 (8.9–9.4)	8.2 (8–8.5)	9.8 (9.6–10.1)	9.7 (9.4–9.9)	9 (8.9–9.1)
4.- Baleares	7.4 (7.2–7.7)	8.2 (8–8.5)	7.2 (7–.4)	8.8 (8.6–9.1)	11.7 (11.5–12)	8.9 (8.8–9.1)
5.- Canarias	2.1 (2–2.2)	3.3 (3.2–3.4)	3.8 (3.7–4)	5.1 (4.9–5.2)	5.7 (5.6–5.9)	4.2 (4.1–4.4)
6.- Cantabria	6.2 (5.9–6.5)	6.2 (5.9–6.5)	5 (4.8–5.3)	6.6 (6.3–6.9)	6.7 (6.4–7)	6.2 (6–6.3)
7.- Castilla y León	10.7 (10.5–10.8)	12.5 (12.3–12.7)	10.5 (10.3–10.7)	11 (10.8–11.2)	12 (11.8–12.1)	11.3 (11.3–11.4)
8.- Castilla-La Mancha	10.9 (10.6–11.1)	12.4 (12.2–12.6)	7.3 (7.1–7.5)	8.2 (8.1–8.4)	7.6 (7.5–7.8)	9.1 (9–9.2)
9.- Cataluña	7.8 (7.7–7.9)	8.5 (8.4–8.6)	7.7 (7.6–7.8)	11.3 (11.2–11.4)	14.8 (14.7–14.9)	10.3 (10.3–10.4)
10.- Valencia	4.3 (4.2–4.4)	5.4 (5.3–5.4)	5.7 (5.6–5.8)	9.1 (9–9.2)	10.9 (10.8–11)	7.4 (7.3–7.4)
11.- Extremadura	6.2 (6–6.4)	10.2 (10–10.5)	8.21 (7.9–8.4)	10.5 (10.2–10.7)	12.1 (11.9–12.4)	9.7 (9.6–9.8)
12.- Galicia	5.9 (5.8–6)	6.9 (6.7–7)	5.2 (5.1–5.3)	7 (6.8–7.1)	9.6 (9.4–9.7)	7 (6.9–7.1)
13.- Madrid	6.5 (6.4–6.6)	7.5 (7.4–7.6)	6.8 (6.8–6.9)	9.6 (9.5–9.7)	10.9 (10.7–10.9)	8.2 (8.2–8.3)
14.- Murcia	7.7 (7.5–8)	9.3 (9.1–9.6)	9 (8.7–9.2)	12.1 (11.8–12.4)	11.9 (11.7–12.2)	10.2 (10.1–10.3)
15.- Navarra	12.8 (12.4–13.3)	7.7 (7.4–8)	5 (4.7–5.2)	5.2 (4.9–5.5)	4.2 (4–4.4)	6.7 (6.5–6.8)
16.- País Vasco	6.8 (6.7–7)	7.8 (7.6–7.9)	6.5 (6.4–6.7)	9.3 (9.2–9.5)	11.6 (11.4–11.7)	8.6 (8.5–8.6)
17.- La Rioja	8.1 (7.6–8.6)	9.9 (9.4–10.5)	7.6 (7.2–8)	9.4 (8.9–9.9)	11.8 (11.3–12.3)	9.5 (9.3–9.7)
18.- Ceuta	8.6 (7.6–9.6)	8.5 (7.5–9.5)	4.5 (3.9–5.2)	7 (6.2–7.9)	7.3 (6.5–8)	7.1 (6.8–7.5)
19.- Melilla	4.9 (4.1–5.7)	5.8 (5–6.7)	4.9 (4.2–5.6)	6 (5.3–6.9)	5.6 (5–6.3)	5.5 (5.2–5.8)
Total	6.9 (6.8–6.9)	7.9 (7.9–7.9)	6.8 (6.8–6.8)	9.1 (9–9.1)	10.7 (10.6–10.7)	8.4 (8.4–8.4)

**Figure 1.**
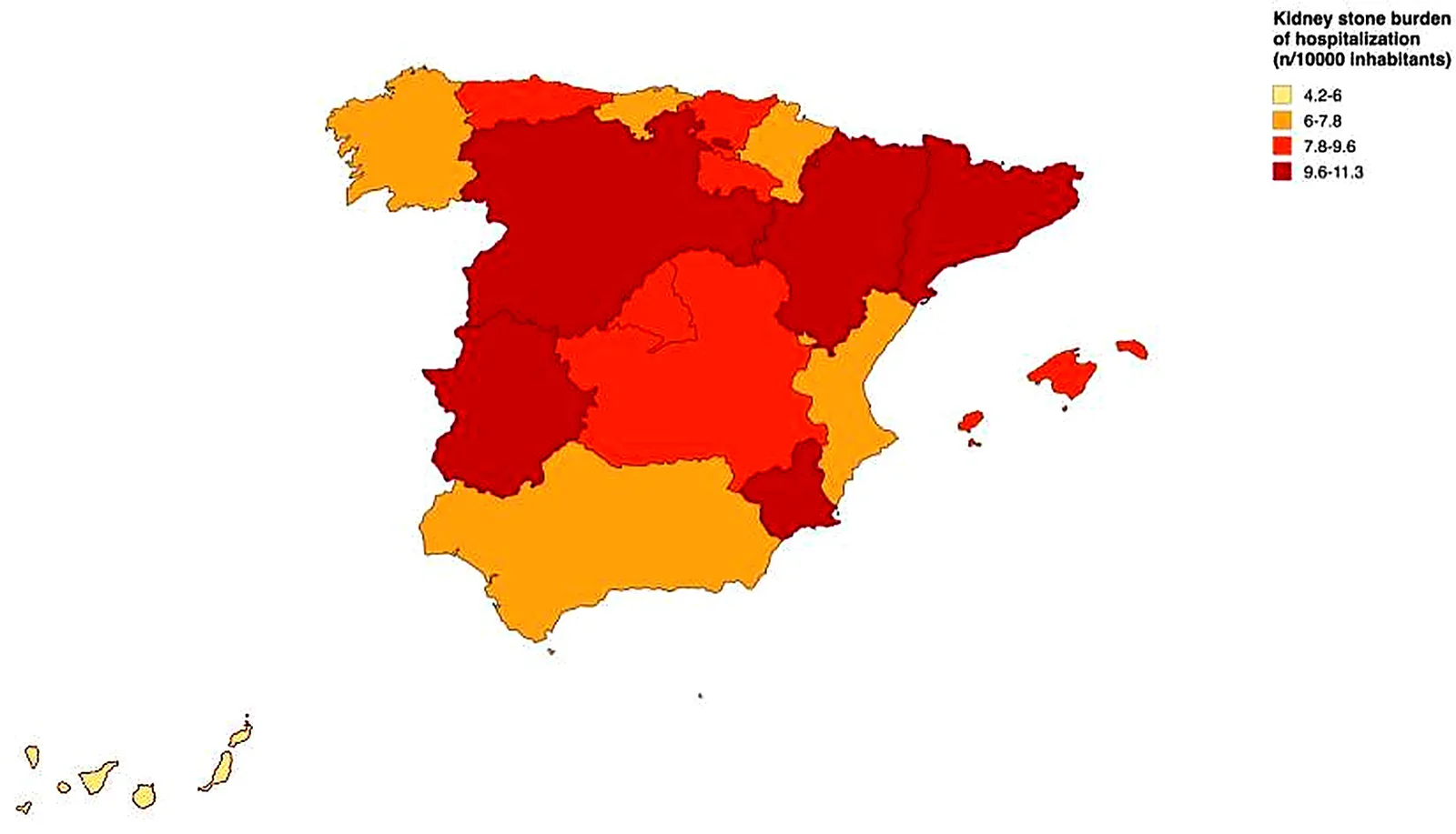
Heat map of hospitalization burden of kidney stones. Annual average from 1997–2022.

Since 1997, the hospitality burden for urinary stone disease has followed a significant increasing trend, with an annual percentage change of 2.14 (CI95%: 0.89–3.4), being higher in men 9.6 (CI95%: 9.6–9.7) than in women 8.2 (CI95%: 8.2–8.3) ( Figure 2).

**Figure 2.**
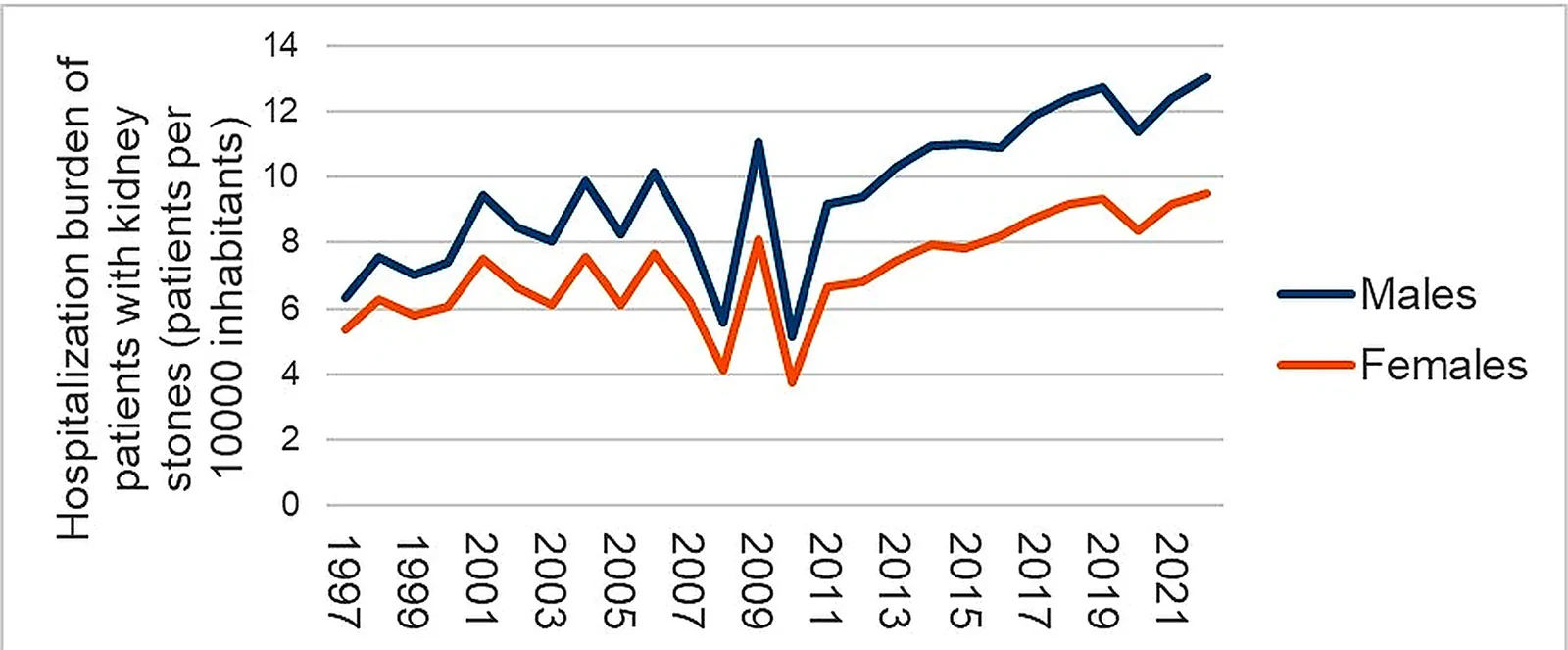
Annual rates of hospitalization burden of patients coded with kidney or ureteral stones. Annual average from 1997–2022. Comparison of male (blue line) and female rates (red line). APC: 2.14 (CI95%: 0.89–3.4).

### Influence of temperature, humidity, radiation and evaporation on hospitality burden of urinary stone disease

To evaluate the influence of climatic factors on the hospitality burden of lithiasis, a correlation test was performed between annual average temperatures and the rates per 10000 inhabitants per region. Humidity was the only factor that reached statistical significance (r = −0.26 (CI95%: −0.26- -0.26; p < 0.001). Temperature, radiation, and evaporation did not reach statistical significance ( Figure 3).

**Figure 3.**
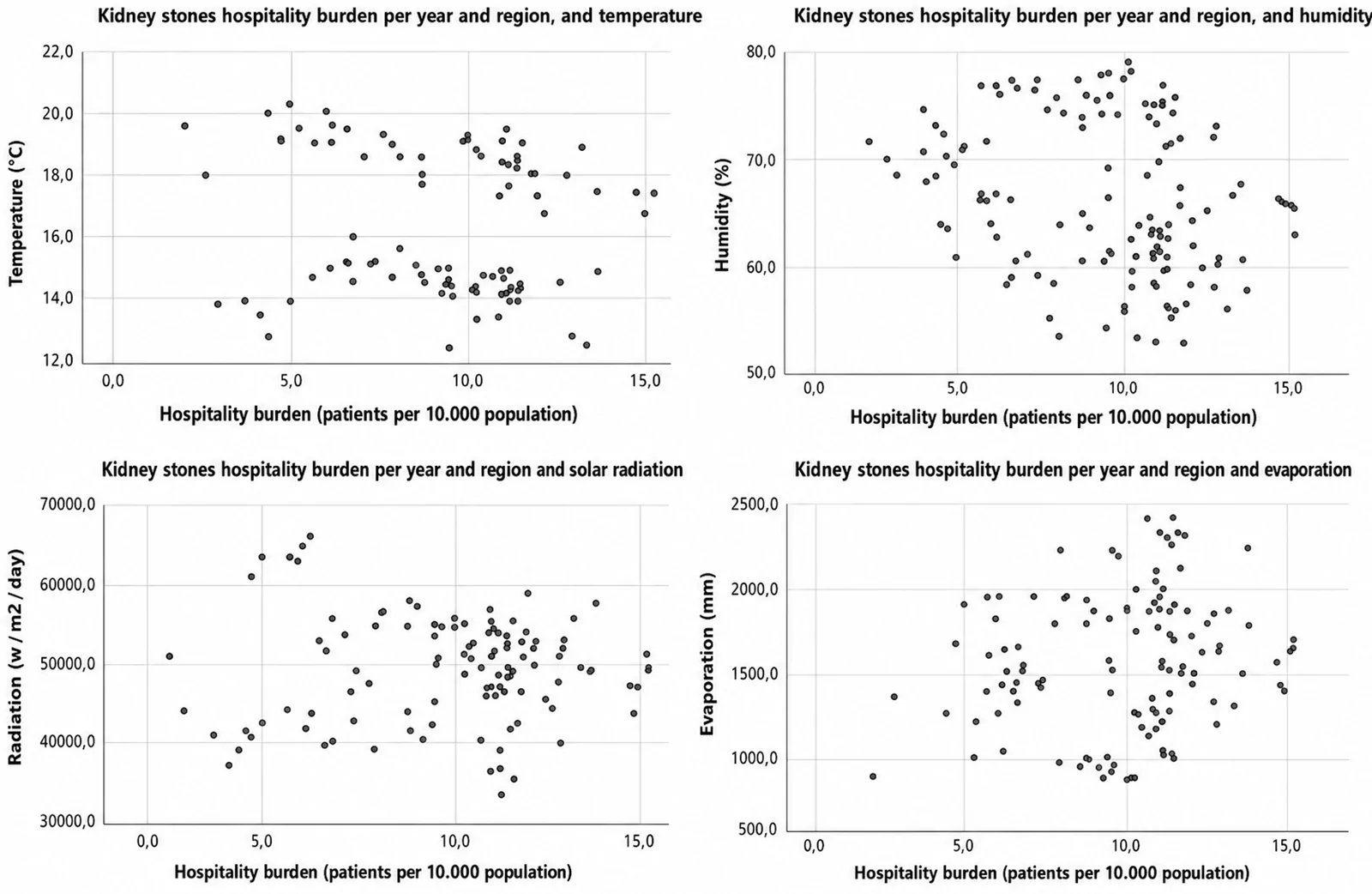
Diagram between the different regions in which are related the overall urolithiasis burden for each average annual temperature of each region (A), humidity (B), radiation (C) and evaporation (D) Bivariate correlation has been performed for the statistical analysis. Only paired values have been represented.

When the average climatic factors during the seven years of follow-up and the incidence of hospitalizations per region annually were studied, no significant correlation was found (see Figure 4). The correlation between the evolution of the kidney stone burden and the average of the different climatic factors per year in the whole country did not reach statistical significance ( Figure 5).

**Figure 4.**
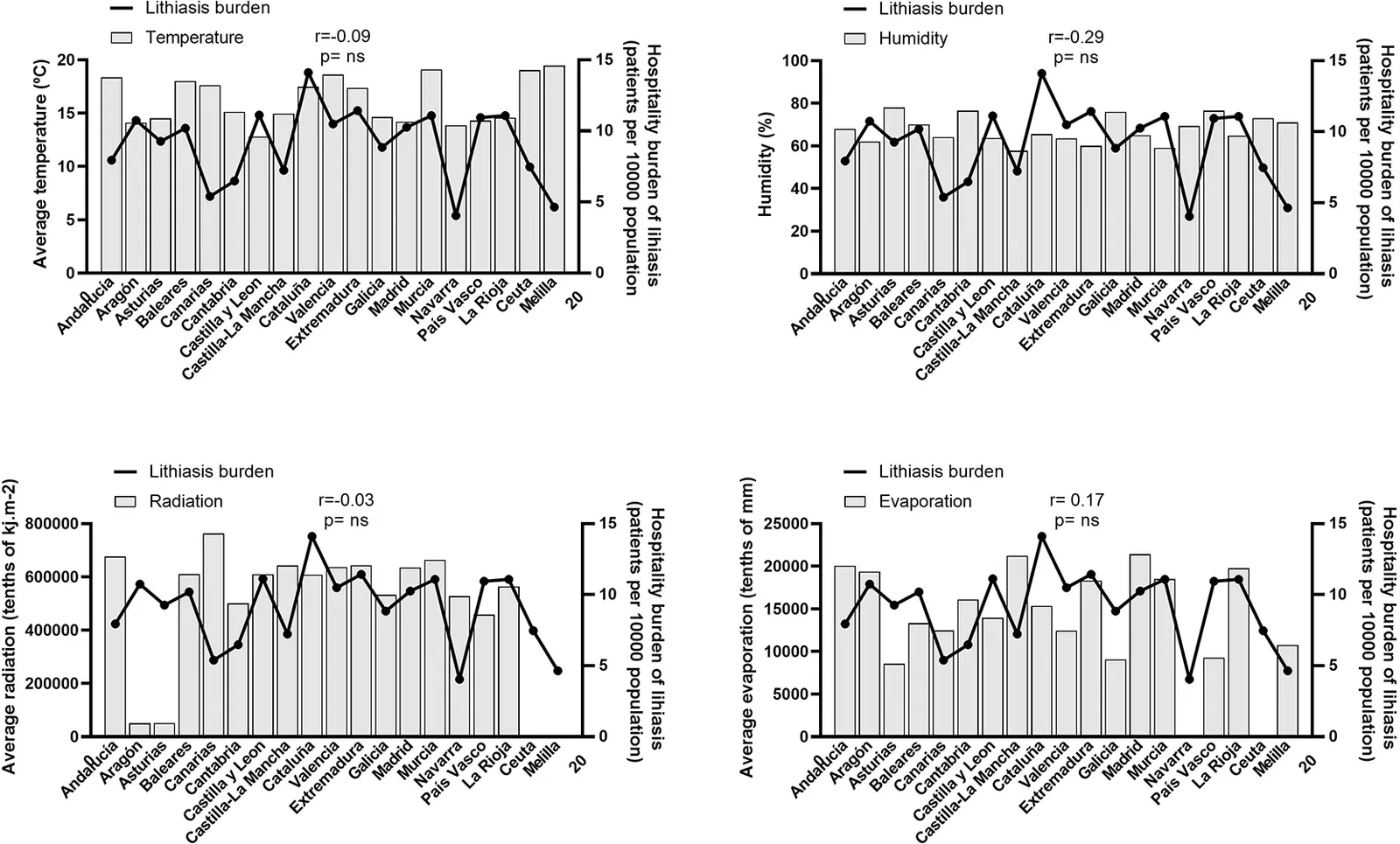
Diagram between the different regions in which are related the overall urolithiasis burden (black line) to average temperature, humidity, radiation or evaporation (grey bars). Bivariate correlation has been performed for the statistical analysis.

**Figure 5.**
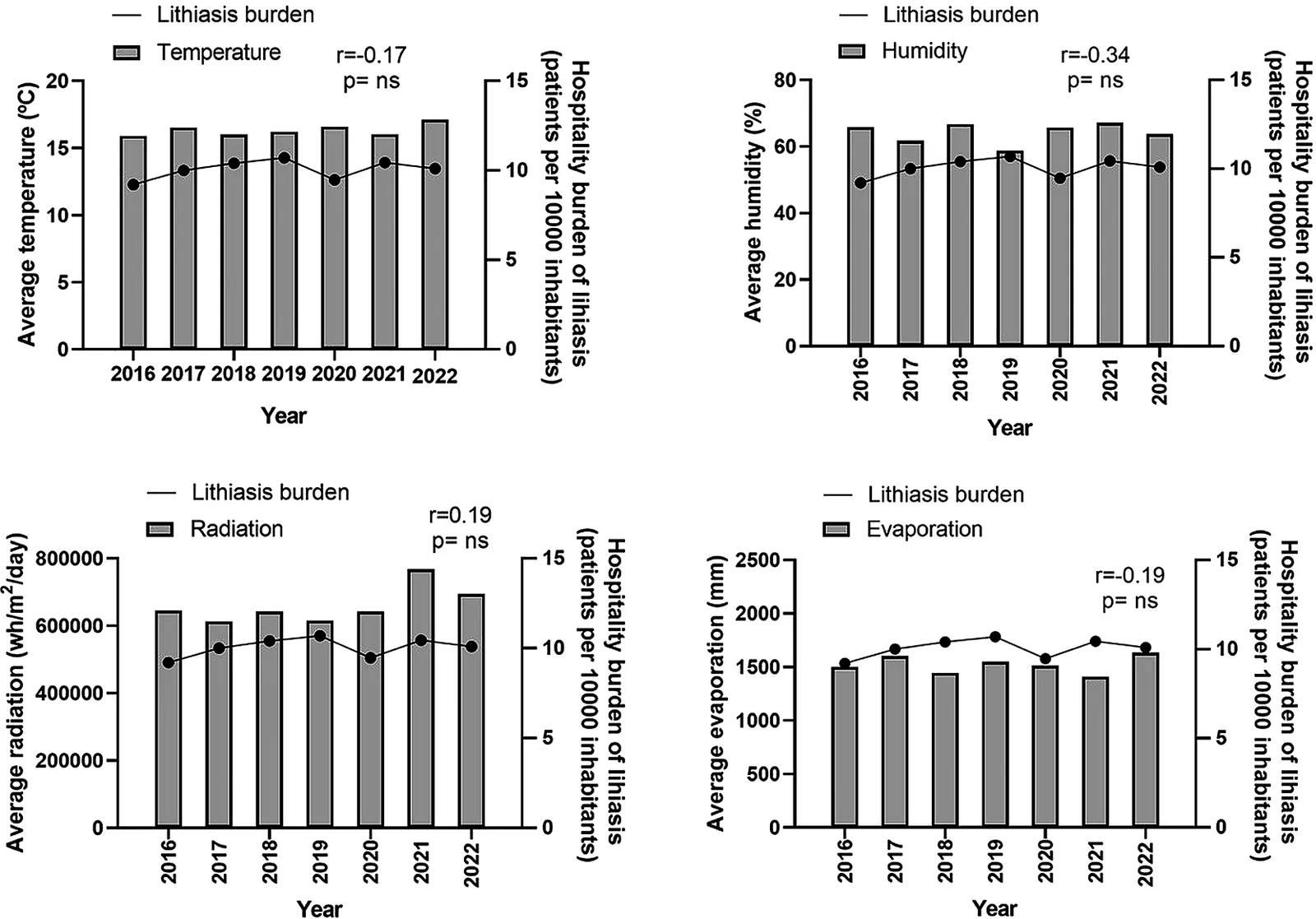
The 6-year period diagram in which are related the overall urolithiasis burden (black line) is related to average temperature, humidity, radiation or evaporation (bars). Bivariate correlation has been performed for the statistical analysis.

Linear regression was used to establish if temperature and humidity could act as confounding factors. Humidity was kept as an independent significant inverse factor for the prevalence of lithiasic hospitalizations (B = -0.12, CI95%: −0.21- -0.04, p = 0.005); however, temperature did not reach statistical significance in the multivariate analysis.

### Influence of the season on hospitality burden of kidney stone disease

To study the influence of season on hospital admissions, the crude rates of each season were compared, and odds ratios were calculated, with winter as the reference category. Spring showed a slight but statistically significant rise OR: 1.1 (1–1.1). This situation was observed in almost every region. Autumn had the lowest hospitalization rate, although no statistical significance was achieved (see Figure 6, Tables 2 and 3).

**Figure 6.**
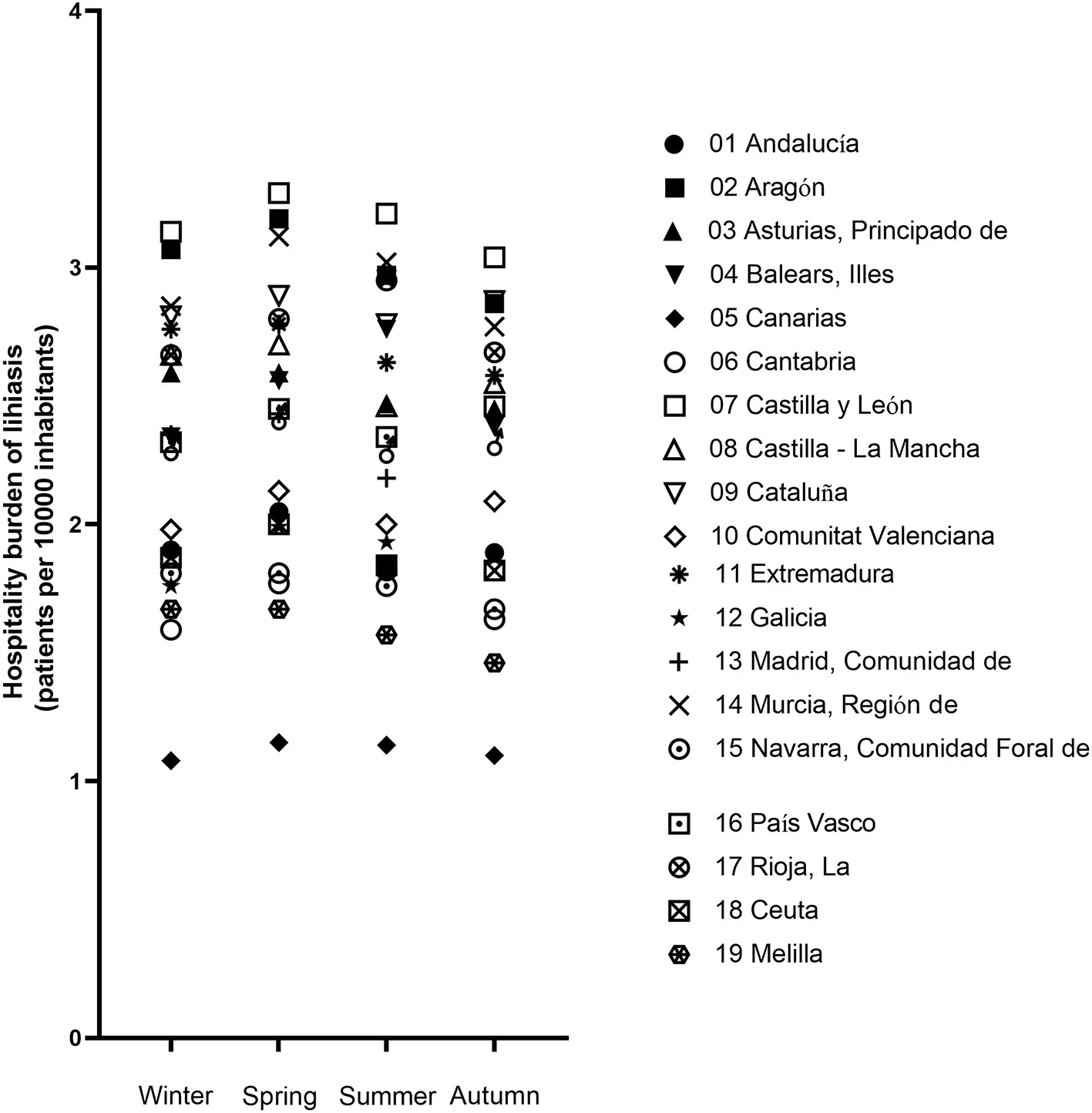
Season rates of hospitalization burden of urolithiasis between 1997 and 2022 stratified by region.

**Table 3.** Season rates of hospitalization burden of urolithiasis between 1997 and 2022 stratified by region. Data are presented as a rate (95% CI) per 10000 inhabitants in the graph, and the odds ratio (95% CI) is the reference for the first category (winter).

Region	Winter	Spring	Summer	Autumn	p
Andalucía	1 (1–1)	1.1 (1.1–1.1)	1 (1–1)	1 (1–1)	<0.001
Aragón	1 (1–1)	1 (1–1)	1 (0.9–1)	0.9 (0.9–1)	<0.001
Asturias	1 (1–1)	1 (1–1)	1 (0.9–1)	0.9 (0.9–1)	<0.001
Baleares	1 (1–1)	1.1 (1.1–1.1)	1.2 (1.1–1.2)	1 (1–1.1)	0.02
Canarias	1 (1–1)	1.1 (1–1.1)	1.1 (1–1.1)	1 (1–1.1)	0.38
Cantabria	1 (1–1)	1.1 (1–1.2)	1.1 (1–1.2)	1 (1–1.1)	0.25
Castilla y León	1 (1–1)	1 (1–1.1)	1 (1–1)	1 (1–1)	<0.001
Castilla-La Mancha	1 (1–1)	1 (1–1)	0.9 (0.9–1)	1 (0.9–1)	<0.001
Cataluña	1 (1–1)	1 (1–1)	1 (1–1)	1 (1–1)	0.30
Valencia	1 (1–1)	1.1 (1–1.1)	1 (1–1)	1.1 (1–1.1)	<0.001
Extremadura	1 (1–1)	1 (1–1)	1 (0.9–1)	0.9 (0.9–1)	<0.001
Galicia	1 (1–1)	1.1 (1.1–1.2)	1.1 (1.1–1.1)	1.1 (1–1.1)	<0.001
Madrid	1 (1–1)	1 (1–1.1)	0.9 (0.9–0.9)	1 (1–1)	0.53
Murcia	1 (1–1)	1.1 (1.1–1.1)	1.1 (1–1.1)	1 (0.9–1)	0.01
Navarra	1 (1–1)	1 (0.9–1.1)	1 (0.9–1)	0.9 (0.9–1)	0.002
País Vasco	1 (1–1)	1.1 (1–1.1)	1.(1-1)	1.1 (1–1.1)	0.002
La Rioja	1 (1–1)	1.1 (0.9–1.1)	1.1 (1–1.2)	1 (0.9–1.1)	0.51
Ceuta	1 (1–1)	1.1 (0.9–1.2)	1 (0.9–1.1)	1 (0.8–1.1)	0.51
Melilla	1 (1–1)	1.1 (0.9–1.2)	0.9 (0.8–1.1)	0.9 (0.7–1)	0.07
Total	1 (1–1)	1.1 (1–1.1)	1 (1–1)	1 (1–1)	<0.001

## Discussion

This study shows a significant rising trend in kidney stone hospitality burden from 1997 to 2023, with significant differences between the regions in Spain, although apparently slightly associated with climatic or seasonal factors.

Several epidemiological studies have confirmed the increasing prevalence of kidney stones in recent decades. ^11^ Indeed, it has been reported that in addition to this rising trend, the male predominance of nephrolithiasis decreases from 1.3:1 to 1.2:1. ^12^ Increased salt and protein consumption and the increasing prevalence of metabolic syndrome have been associated with higher rates of kidney stones in developed countries. ^11, 13, 14^ It has recently been shown that the increasing burden of kidney stone hospitalizations is linked to rising obesity rates in Spain, resulting in a higher financial burden for healthcare systems. ^15^

Several factors have been related to this association, oxidative stress has been reported as a common link, underlying the exacerbated inflammatory response under conditions of both obesity and hyperoxaluria in experimental rat models. ^16^

Obesity and kidney stones relationship has been confirmed from an epidemiologic point of view through a meta-analysis in which obese patients show a higher risk of developing stones (OR = 1.35; CI95%: 1.2–1.5, p < 0.001). ^17^

However, in addition to dietary factors, other factors have also been pointed out as being responsible for the increasing prevalence observed in the last decades. Global warming and an increase in ambient temperature have been associated with these rising trends. It is postulated that higher insensible losses from sweat increase result in an increase in vasopressin secretion, lower urine volume, urinary concentration, and higher urinary supersaturation of ions. ^18^

Several studies have attempted to elucidate whether climate change can predict variations in the number of hospitalizations due to kidney stones. Temperature and humidity are widely associated with the incidence of kidney stones. ^5, 19^ However, the influence of evaporation and solar radiation on the appearance of kidney stones has been poorly studied.

Although patients with kidney stone events are generally hospitalized when they become symptomatic, and other factors such as kidney stone detachment are implied in this issue, supersaturation of urine solutes and urinary crystallization are unavoidable factors to consider. Temperature and evaporation directly influence evaporative body water loss through sweating and respiration. The resultant low urine volume increases urine supersaturation, leading to stone growth in susceptible patients. Evaporative water loss is determined by the difference in water vapor concentration between the body surface and ambient air. ^20^

Some studies have reported that the incidence of kidney stones is higher in warmer places when associated with reduced water intake ^2, 21^; however, the combined effect of temperature and relative humidity better predicts kidney stone presentation. Recent studies have pointed out the better role of wet-bulb temperature than dry-bulb temperatures in kidney stone incidence. ^5^ The wet-bulb temperature is defined as the lowest temperature that can be reached under current ambient conditions by the evaporation of water only. ^22^ Therefore, the wet-bulb temperature will be higher when the humidity is higher because the evaporation will be lower.

In our study, the mean annual temperature by region was not associated with kidney stone hospitality burden. Although differences have been observed between different regions, regions with higher temperatures do not have the highest kidney stone hospitalizations. Classical studies linking temperature with kidney stone prevalence have been conducted in large countries with broad variations in temperature. ^2, 23^ Spain is a small country with a Mediterranean climate and narrow temperature variations.

However, relative humidity is inversely related to kidney stone hospitalization. Our data are similar to those reported by Abreu et al., ^23^ in which a significant negative relationship was also found between relative humidity and nephrolithiasis hospitalizations. Other reports have also highlighted the importance of humidity in the development of kidney stones, suggesting that the risk of renal calculus formation is better predicted by the combination of temperature and renal humidity. ^5^ In our case, when considering temperature and humidity, humidity had an inverse significant relationship with the prevalence of lithiasis hospitalizations.

In Spain, one study has reported a positive relationship between renal lithiasis and average annual temperatures, even though the study was limited to Andalusian populations between 40 and 65 years old and was developed through telephone surveys. ^8^ At this point, we consider that the study is not comparable to ours, either by methodology or reference population.

The roles of sun exposure and vitamin D in kidney stone formation have not yet been properly defined. The first references that suggested this relationship were based on measuring the urine composition of soldiers before and after their transfer to a tropical climate. ^24^ Theoretically, sunlight exposure leads to an increased production of vitamin D, which is converted to 1,25-dihydroxyvitamin D in the kidney to increase the intestinal absorption of dietary calcium and prompts the kidney to eliminate more calcium. ^25^ Increased intestinal calcium absorption increases intestinal oxalate absorption. ^26^ Thus, hypercalciuria and hyperoxaluria are conducive to stone production. ^27^ However, a large, population-based, randomized, controlled study has shown that vitamin D supplementation is not significantly associated with either hypercalcemia or nephrolithiasis. ^28^ Therefore, sunlight exposure does not have a significant impact on calcium metabolism in the human body. Our study did not find a significant relationship between solar radiation, which should be related to sun exposure, and hospitality burden due to kidney stones. At this point, it is remarkable that traditionally sunny regions, such as the Canary Islands, Melilla, and Andalucía, showed the lowest impact on kidney stone hospitalization.

Several studies have examined the relationship between climatic seasons and the incidence of kidney stones and urine composition. Results regarding the incidence of nephrolithiasis are controversial. A higher incidence of renal colic has been reported in the emergency department during the summer months, especially in older people. ^29, 30^ On the other hand, other authors did not find seasonal variation in the incidence of stones removed, although in the same study, they reported a 50% higher stone passage in the summer with a significant increase in the saturation of urine with calcium oxalate. ^31^

The hottest months of the year are associated with changes in urine composition consisting of super-saturation of calcium oxalate and calcium phosphate. ^19, 32^ In our study, there was a slight but significantly higher incidence of lithiasis hospitalizations during spring and summer than in winter and autumn. These data seem to agree with those of other studies that showed a higher incidence of stone episodes during the hottest months of the year.

This study had some limitations. First, underestimation of climate exposure is possible because the analysis is performed with average arithmetical measures, without considering the maximum or minimal figures. This issue doesn’t represent the 24 hours development, especially in continental areas where the parameter variability could be higher. Additionally, we do not know which individuals have access to resources that reduce the effects of heat and cold.

Additionally, no techniques have been applied to correct temporal or neighboring. Pearson assume that every point data is independent and identically distributed. Hospitalizations of neighboring areas or years inherently carry the memory of the others and could display significant correlations. In our case, the only correlation documented has been the humidity wich has been supported by the literature.

On the other hand, Spain is a relatively small country when compared with other countries in which studies have reported changes in kidney incidence associated with climate. Spain enjoys a Mediterranean climate in most of its geography, which could also act as a confounding factor. In addition, MDBS does not include emergency visits or ambulatory procedures such as lithotripsy; therefore, hospitalization burden should not be considered as a measure of incidence.

Either way, our study confirms a significant rising trend of hospitalizations due to kidney stone disease in the last decades, with significant differences between regions inside the Spanish country that could be partially explained by changes in humidity values measured by the Spanish meteorology agency. Temperature, solar radiation, and evaporation do not seem to influence kidney hospitalizations.

## Ethics approval and consent to participate

No ethical approval was needed for this study because it was based on secondary analysis of the data obtained from the CMBD (Minimum Basic Data Set) of the Ministry of Health, Social Services and Equality of Spain. The data were completely anonymous, and this study uses data with no identifiable information on the survey participants. The Ministry of Health Social Services and Equality of Spain (Subdirección General del Instituto de Información) gave us administrative permission to access and use the data on which this study is based.

## Data Availability

Data are available under the terms of the Creative Commons Zero “No rights reserved” data waiver (CC0 1.0 Public domain dedication). The article follows the open data policy. Data can be accessed 10.5281/zenodo.18908586. ^33^
