# Supplementary material for: The effect of geography and climatic factors on the hospitality burden due to kidney stones in Spain 1997-2022
Source: Open Res Eur. 2026 Jul 15;6:116. Originally published 2026 Apr 16. [Version 2] doi: 10.12688/openreseurope.23359.2 (PMC13560556; doi:10.12688/openreseurope.23359.2)
Supplement: Peer review history

# Reviewer report: Reviewer response for version 2

**DOI:** 10.21956/openreseurope.26346.r78603

**Published:** 2026-08-25

**Authors:** Mohemid Maddallah Al-Jebouri

**Affiliations:** 1 Al-Qalam University, Kirkuk, Iraq

Abstract:

 It is clear and reasonably presented showing the outlines of the article.

 Introduction:

 It covers most of the relevant literature with clear aim of the work showed.

 Methodology:

 Data collection, gathering, and designed is well treated diving a good conclusive and clear image for speculation and assessment of kidney stone distribution among population under study. However, all the retrospective studies have their weak points by missing of other demographic factors that might affect kidneys leading to urolithiasis.

 Statistical analysis:

 The data are well treated using MANOVA,ANOVA and other related statistical analyses, but for simulation purposes particularly for such large number of data I think using of a mathematical analysis such as dynamics and phase portraits is very useful for assessment and for future planning and controlling.

 Results:

 All data, tables and figures are well presented and easy to follow.

 Discussion:

 Most relevant literature is covered.

 References:

  Some useful articles are missed.

 Language:

 The article is well-written.

Is the study design appropriate and does the work have academic merit?

No

Is the work clearly and accurately presented and does it cite the current literature?

Yes

If applicable, is the statistical analysis and its interpretation appropriate?

Yes

Are all the source data underlying the results available to ensure full reproducibility?

Yes

Are the conclusions drawn adequately supported by the results?

Yes

Are sufficient details of methods and analysis provided to allow replication by others?

Yes

Reviewer Expertise:

NA

I confirm that I have read this submission and believe that I have an appropriate level of expertise to confirm that it is of an acceptable scientific standard, however I have significant reservations, as outlined above.

# Reviewer report: Reviewer response for version 2

**DOI:** 10.21956/openreseurope.26346.r77443

**Published:** 2026-07-23

**Authors:** Juan Antonio Galán Llopis

**Affiliations:** 1 General University Hospital Dr Balmis, Alicante, Spain

The current submission is clear, easy to read, appropriately presented and cites the current literature. It consists of a retrospective observational study using the reliable Minimum Basic Data System (MBDS) public registry of the Spanish Ministry of Health and Social Services. It gathers information about hospitalizations due to kidney across Spain during a 25-year period. Data related to climatic conditions have also been obtained from the also official Spanish Meteorological Agency, so they are easily available.

 The study confirms a significant rising trend of KSD hospitalizations with significant differences across different regions, which could be partially explained only by changes in humidity values as the other studied conditions do not seem to have any influence on hospitalizations due to kidney stones.

 The submission has academic merit as it relates to the specific climatic conditions affecting stone disease related hospitalizations in a country where not many deep differences in climate exist apart from very few examples.

 After the corrections made, the article can, in my opinion, be accepted for final indexing.

Is the study design appropriate and does the work have academic merit?

Yes

Is the work clearly and accurately presented and does it cite the current literature?

Yes

If applicable, is the statistical analysis and its interpretation appropriate?

Yes

Are all the source data underlying the results available to ensure full reproducibility?

Yes

Are the conclusions drawn adequately supported by the results?

Yes

Are sufficient details of methods and analysis provided to allow replication by others?

Yes

Reviewer Expertise:

NA

I confirm that I have read this submission and believe that I have an appropriate level of expertise to confirm that it is of an acceptable scientific standard.

# Reviewer report: Reviewer response for version 1

**DOI:** 10.21956/openreseurope.25272.r73909

**Published:** 2026-05-27

**Authors:** Darsy Darssan

**Affiliations:** 1 School of Public Health, The University of Queensland (Ringgold ID: 1974), Herston, Queensland, Australia

This study uses geographical and environmental data from a large national hospitalisation dataset, comprising 980,701 episodes over 26 years. A major strength is the use of the Spanish Minimum Basic Data Set (MBDS), which covers approximately 98% of all hospitalisations across Spain.

 Here are my specific review comments and feedback:

**Methods**

 1. Study design section first paragraph:

 The statement on secondary analysis is unnecessary:

         a. It is redundant for a study using anonymised administrative registry data, as this is standard practice and does not require individual consent.

         b. The sentence about minor patients and legal guardian permission is not relevant. I did not come across any mention of paediatric cases or the study population's age range.

 2. Study design section, two web links:

         The two links,

 a. https://www.ine.es/jaxiT3/Tabla.htm?t=56934 and

 b. https://www.aemet.es/es/datos_abiertos should be added to the reference list and cited here.

 3. Study design section, last paragraph:

 ‘climatic conditions’ is not the most appropriate term. Climatic conditions usually refer to long-term climate patterns (typically over 30 years). Since you are using yearly Average temperature, humidity, radiation, and evaporation, change the term to ‘meteorological variables’ or ‘weather parameters’

 4. Study design section, the last line: It is unclear and mixes data preparation with analysis. Delete or change, “…., *and have been related to the rates of urinary stone disease*.” Do you mean that these metrological variables were then analysed in relation to regional hospitalisation rates for urinary stone disease?

 5. Statistical analysis section first paragraph:

        a. Is the Kidney stone hospitalisation rate age-standardised? Since you have data for the longer-term trend, this is important.

        b. per 10000 inhabitants

            i. Change to per 10,000 population. If you don’t like the term ‘population’, please use residents. The term ‘inhabitants’ is odd and old-fashioned. The most standard and widely used term in epidemiology is ‘per 10,000 population’

            ii. Where did you get the population denominator? This is not explained in the previous ‘study design’ section. Must reference the source and cite it or provide the population estimation for each region. Is it the mid-year population?

      c. 95% confidence interval

      Must specify for what estimate you provide the confidence interval. Is it for the APC (Annual Percentage Change), that is, for each individual year segment? Or Average Annual Percentage Change (AAPC), that is for the overall trend? Or both APC and AAPC?

      d. ‘joint-point’ regression

           i. Not the right term, joinpoint regression without a ‘t’ is the correct term. Join-point regression (with hyphen and without ‘t’) is sometimes acceptable. Piecewise linear regression, segmented regression and change-point regression are other widely used terms.

          ii. Specify the minimum and maximum number of joinpoints (model parameters) used in your modelling

          iii. State the method used to select the final model, for example, the permutation test or the Bayesian Information Criterion

          iv. Since you calculated the percentage change, it is highly likely you should have used a log transformation of your rates, which is a standard practice; if so, please specify that you have log-transformed your rates.

 6. Statistical analysis section, second paragraph:

        a. “…a correlation test was performed between the average measures and hospitalisation burden from 2016 to 2022 per year and region.”

             i. There is a critical inconsistency between the stated data availability (1997–2022) and the analytical period (2016–2022). Why were the years 1997 – 2015 excluded?

             ii. What correlation test was used? Must specify this, it is likely Pearson or Spearman?

            iii. Temporal autocorrelation: For example, the hospitalisations in 2021 are not independent of 2020; temporal autocorrelation is not considered in this work. Am I right? This is a major concern if you just used Pearson or Spearman correlation.

            iv. Neighbouring regions (Ceuta) share a similar climate and population. Spatial autocorrelation is not taken into account in this work. Another concern you will need to address. I looked for the discussion of these limitations in your discussion section. There are figures and tables (results) in the discussion section, but no discussion of these limitations.

      b. Must use a method to visualise how the metrological variables in the study vary across Spain.

          Choropleth maps are essential in the results section, and their use should be written in the methods section, stating that such visualisation is used. Hospitalisation is shown in Figure 1, but the methods section needs a sentence on the visualisation of temperature, humidity, etc. If there are too many figures, some of them go to the supplementary file.

 7. Multiple testing

          Testing temperature, humidity, radiation and evaporation against hospitalisation burden across multiple regions and years creates multiple comparisons. Adjustment for multiple comparisons should be made.

**Results**

 Figures 3,4 and 5 are unclear and not easily interpretable.

 1. Figure 3, did you try interpreting the correlation coefficients you presented at the top of each sub-figure? They are meaningless.

 2. The legends have years and regions, but readers cannot see these clearly in the figure

 3. These are not good figures; please find alternative ways to present.

 4. Figure 4, the y-axis on the right-hand side has changed the hospital burden per 100,000; earlier, it was per 10,000. How come in Figure 3 they varied between 2 and 16 when reported per 10,000, but here in Figure 4 they vary between 25,00 and 150,00. Is there something wrong with the calculation?

Is the study design appropriate and does the work have academic merit?

Partly

Is the work clearly and accurately presented and does it cite the current literature?

Partly

If applicable, is the statistical analysis and its interpretation appropriate?

Partly

Are all the source data underlying the results available to ensure full reproducibility?

Partly

Are the conclusions drawn adequately supported by the results?

Partly

Are sufficient details of methods and analysis provided to allow replication by others?

Partly

Reviewer Expertise:

Spatial Health and Environmental Impact Modelling

I confirm that I have read this submission and believe that I have an appropriate level of expertise to confirm that it is of an acceptable scientific standard, however I have significant reservations, as outlined above.

# Response

**Published:** 2026-06-14

**Authors:** JAVIER SAENZ MEDINA

**Affiliations:** Urology, Ramon y Cajal Hospital Urology Department, Madrid, Community of Madrid, Spain

Thank you very much for your valuable comments and for your contribution to the paper, I really appreciate them. Below are responses for all of them.  

 1.- Study design section first paragraph:

 The statement on secondary analysis is unnecessary:

         a. It is redundant for a study using anonymised administrative registry data, as this is standard practice and does not require individual consent.

         b. The sentence about minor patients and legal guardian permission is not relevant. I did not come across any mention of paediatric cases or the study population's age range. **Response: **Effectively the sentence is redundant. It was not included in the original draft, but it was included in response to the request of editorial team. Both sentences are deleted.

     2. Study design section, two web links:

         The two links,

 a. https://www.ine.es/jaxiT3/Tabla.htm?t=56934 and

 b. https://www.aemet.es/es/datos_abiertos should be added to the reference list and cited here.  

**Response: **Bot citations are included in bibliography.

  3. Study design section, last paragraph:

 ‘climatic conditions’ is not the most appropriate term. Climatic conditions usually refer to long-term climate patterns (typically over 30 years). Since you are using yearly Average temperature, humidity, radiation, and evaporation, change the term to ‘meteorological variables’ or ‘weather parameters’

**Response: ** The term has been changed. Thank you very much.  

 4. Study design section, the last line: It is unclear and mixes data preparation with analysis. Delete or change, “…., *and have been related to the rates of urinary stone disease*.” Do you mean that these metrological variables were then analysed in relation to regional hospitalisation rates for urinary stone disease?

**Response: ** Thank you very much. Clarification is performed in the text.

     5. Statistical analysis section first paragraph:

        a. Is the Kidney stone hospitalisation rate age-standardised? Since you have data for the longer-term trend, this is important.

        b. per 10000 inhabitants

            i. Change to per 10,000 population. If you don’t like the term ‘population’, please use residents. The term ‘inhabitants’ is odd and old-fashioned. The most standard and widely used term in epidemiology is ‘per 10,000 population’

            ii. Where did you get the population denominator? This is not explained in the previous ‘study design’ section. Must reference the source and cite it or provide the population estimation for each region. Is it the mid-year population?

      c. 95% confidence interval

      Must specify for what estimate you provide the confidence interval. Is it for the APC (Annual Percentage Change), that is, for each individual year segment? Or Average Annual Percentage Change (AAPC), that is for the overall trend? Or both APC and AAPC?

      d. ‘joint-point’ regression

           i. Not the right term, joinpoint regression without a ‘t’ is the correct term. Join-point regression (with hyphen and without ‘t’) is sometimes acceptable. Piecewise linear regression, segmented regression and change-point regression are other widely used terms.

          ii. Specify the minimum and maximum number of joinpoints (model parameters) used in your modelling

          iii. State the method used to select the final model, for example, the permutation test or the Bayesian Information Criterion

          iv. Since you calculated the percentage change, it is highly likely you should have used a log transformation of your rates, which is a standard practice; if so, please specify that you have log-transformed your rates.

**Response:** *a. Yes, the rates are age standardized. Clarification is performed in the paper*

  b. *i the term has changed to population*

*b.ii the population denominator Is gotten from National institute of statistics. It is described in the study design.* *c.i 95% confidence interval is provided for the average annual percentage change. It is corrected in the text.* *d.i It is corrected in the text.* d.ii and diii In our model minimum number of joinpoints are 0, and maximum number number of joinpoints are 4. The method used to select the final model was the weighted Bayesian information criterion. Finally, the model recommended was with 0 joinpoints. All these points are included in the statistical analysis description. d.iv Log transformation of rates have been used for to calculate the percentage change, and it has been included in the text.  

 6. Statistical analysis section, second paragraph:

        a. “…a correlation test was performed between the average measures and hospitalisation burden from 2016 to 2022 per year and region.”

             i. There is a critical inconsistency between the stated data availability (1997–2022) and the analytical period (2016–2022). Why were the years 1997 – 2015 excluded?

             ii. What correlation test was used? Must specify this, it is likely Pearson or Spearman?

            iii. Temporal autocorrelation: For example, the hospitalisations in 2021 are not independent of 2020; temporal autocorrelation is not considered in this work. Am I right? This is a major concern if you just used Pearson or Spearman correlation.

            iv. Neighbouring regions (Ceuta) share a similar climate and population. Spatial autocorrelation is not taken into account in this work. Another concern you will need to address. I looked for the discussion of these limitations in your discussion section. There are figures and tables (results) in the discussion section, but no discussion of these limitations.

      b. Must use a method to visualise how the metrological variables in the study vary across Spain.

          Choropleth maps are essential in the results section, and their use should be written in the methods section, stating that such visualisation is used. Hospitalisation is shown in Figure 1, but the methods section needs a sentence on the visualisation of temperature, humidity, etc. If there are too many figures, some of them go to the supplementary file.  

**Response:**

   a.i: No meteorogical data were available from 1997 to 2015. So, for that reason were not included. Clarification has been included in the text. a.ii Pearson correlation test was used.  Clarification has been added to the text. a.iii and iv Effectively temporal and spatial autocorrelation are not considered, so regions and years are considered independently. This state could would display significant correlations which could be spurious. In our case, correlation test has not display significant results in exception for the humidity which has been widely documented in the literature. These points are included in the limitations paragraph. b. Chropleth maps have been included as Figure Supplementary 1.. They are detailed in the methods and the results section.

**Results**

 Figures 3,4 and 5 are unclear and not easily interpretable.

 1. Figure 3, did you try interpreting the correlation coefficients you presented at the top of each sub-figure? They are meaningless.

 2. The legends have years and regions, but readers cannot see these clearly in the figure

 3. These are not good figures; please find alternative ways to present.

 4. Figure 4, the y-axis on the right-hand side has changed the hospital burden per 100,000; earlier, it was per 10,000. How come in Figure 3 they varied between 2 and 16 when reported per 10,000, but here in Figure 4 they vary between 25,00 and 150,00. Is there something wrong with the calculation?  

**Response: ** Figure 3 has been redesigned and the correlation coefficients with confidence intervals and p have been deleted. Figure 4 has been redesigned.

# Reviewer report: Reviewer response for version 1

**DOI:** 10.21956/openreseurope.25272.r72702

**Published:** 2026-05-19

**Authors:** Juan Antonio Galán Llopis

**Affiliations:** 1 General University Hospital Dr Balmis, Alicante, Spain

The current submission is clear, easy to read, appropriately presented and cites the current literature. It consists of a retrospective observational study using the reliable Minimum Basic Data System (MBDS) public registry of the Spanish Ministry of Health and Social Services. It gathers information about hospitalizations due to kidney across Spain during a 25-year period. Data related to climatic conditions have also been obtained from the also official Spanish Meteorological Agency, so they are easily available.

 The study confirms a significant rising trend of KSD hospitalizations with significant differences across different regions, which could be partially explained only by changes in humidity values as the other studied conditions do not seem to have any influence on hospitalizations due to kidney stones.

 The submission has academic merit as it relates to the specific climatic conditions affecting stone disease related hospitalizations in a country where not many deep differences in climate exist apart from very few examples.

 A couple of comments regarding the submission:

 First paragraph on Discussion: “although apparently not associated with climatic or seasonal factors”. Is it right?. Table 3 seems to reflect some differences related to seasons.

 Last paragraph on page 7:” This relationship has also been confirmed from an epidemiologic point of view through a meta-analysis in which an OR of 1.35 (CI95%: 1.2–1.5, p < 0.001) for developing kidney stones in obese participants.” should be rewritten for better understanding.

Is the study design appropriate and does the work have academic merit?

Yes

Is the work clearly and accurately presented and does it cite the current literature?

Yes

If applicable, is the statistical analysis and its interpretation appropriate?

Yes

Are all the source data underlying the results available to ensure full reproducibility?

Yes

Are the conclusions drawn adequately supported by the results?

Yes

Are sufficient details of methods and analysis provided to allow replication by others?

Yes

Reviewer Expertise:

NA

I confirm that I have read this submission and believe that I have an appropriate level of expertise to confirm that it is of an acceptable scientific standard, however I have significant reservations, as outlined above.

# Response

**Published:** 2026-06-14

**Authors:** JAVIER SAENZ MEDINA

**Affiliations:** Urology, Ramon y Cajal Hospital Urology Department, Madrid, Community of Madrid, Spain

Thank you very much for your comments, I really appreciate your contribution,and all your suggestions are included in the new version.
